# Supplementary material for: Pharmacology of Veratrum californicum Alkaloids as Hedgehog Pathway Antagonists
Source: Pharmaceuticals (Basel). 2024 Jan 17;17(1):123. doi: 10.3390/ph17010123 (PMC10821092; doi:10.3390/ph17010123)
Supplement: Supplementary file 1 [file pharmaceuticals-17-00123-s001.zip › pharmaceuticals-2758845-supplementary.pdf]

Supplemental Materials

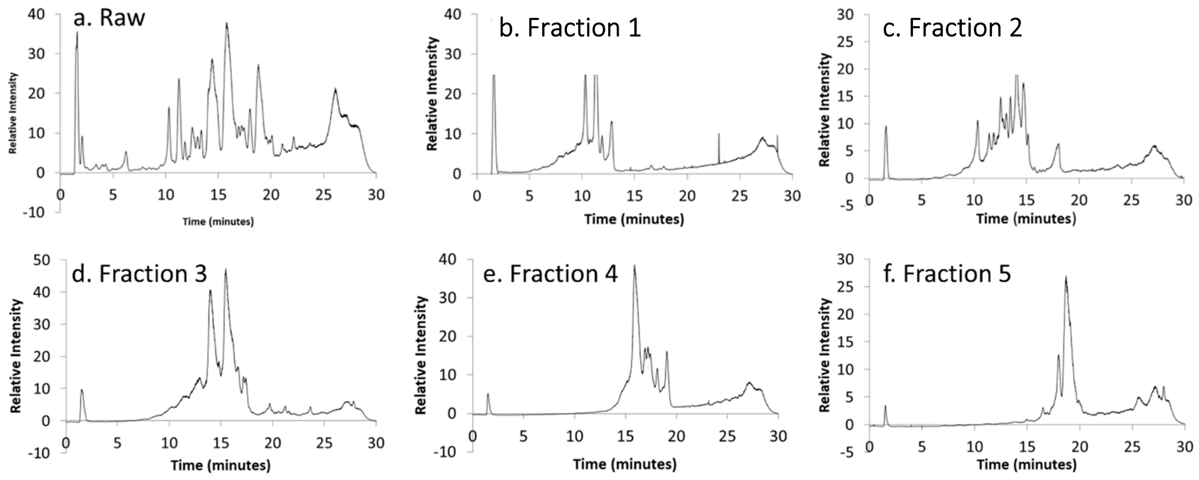

Supplemental Figure S1. Chromatograms of *V. californicum* using HPLC-CAD: a. raw extract, b. fraction 1 (10.75-13.25 minutes), c. fraction 2 (13.25-15.75 minutes), d. fraction 3 (15.75-18.25), e. fraction 4 (18.25-20.75 minutes), and f. fraction 5 (20.75-23.25 minutes).

The solvents and gradient were the same for this method as the one described above, but the flow rate was lowered to 0.3 mL/minute for a Thermo Acclaim 120 C_{18} column (2.1 x 150 mm, 3 µm). The chromatograms of the fractions as captured by the CAD can be seen in **Figure S1 b-f,** where **Figure S1 b** shows fraction 1 (collection time 10.75 to 13.25 minutes), **Figure S1 c** shows fraction 2 (collection time 13.25 to 15.75 minutes), **Figure S1 d** shows fraction 3 (collection time 15.75 to 18.25 minutes), **Figure S1 e** shows fraction 4 (collection time 18.25 to 20.75 minutes), **Figure S1 f s**hows fraction 5 (collection time 20.75 to 23.25 minutes), and **Figure S1 g** shows an overlaid view of each fraction.

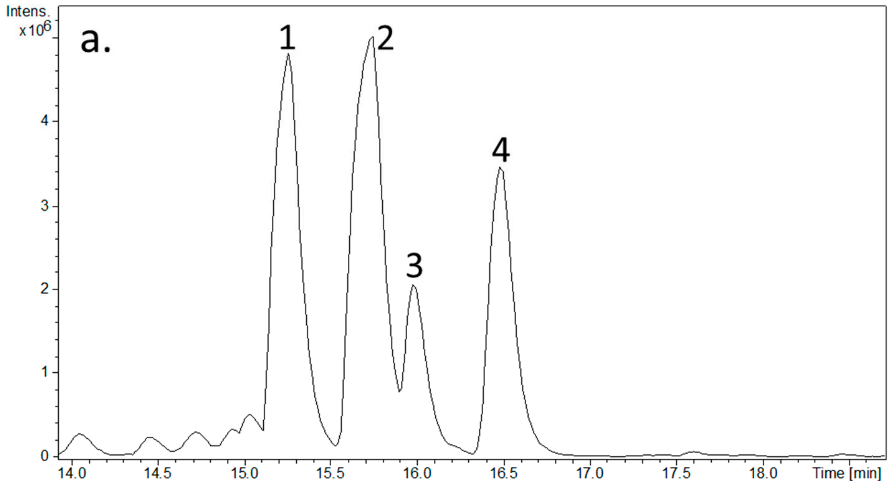

Supplemental Figure S2. Chromatogram of alkaloids present in fraction 1, which corresponds to R_t from 10.75 – 13.25 minutes of crude extract chromatogram.

Fraction 1 contained four alkaloids as shown in **Figure S2.** The peaks appeared to be paired with 1 and 3 sharing nearly identical m/z ratios of 572.3284 and 572.3271 (elution times of 15.3 and 16.0 minutes) and peaks 2 and 4 following the same pattern with m/z ratios of 574.3451 and 574.3428 respectively (elution times of 15.7 and 16.5 minutes). The predicted chemical formulas are $C_{33}H_{49}NO_7$ for peaks 1 and 3 and $C_{33}H_{51}NO_7$ for the peaks 2 and 4. The reasonable identity of these alkaloids is veratrosine for peak 1, cycloposine for peak 2, and isomers of each for peaks 3 and 4.[4]

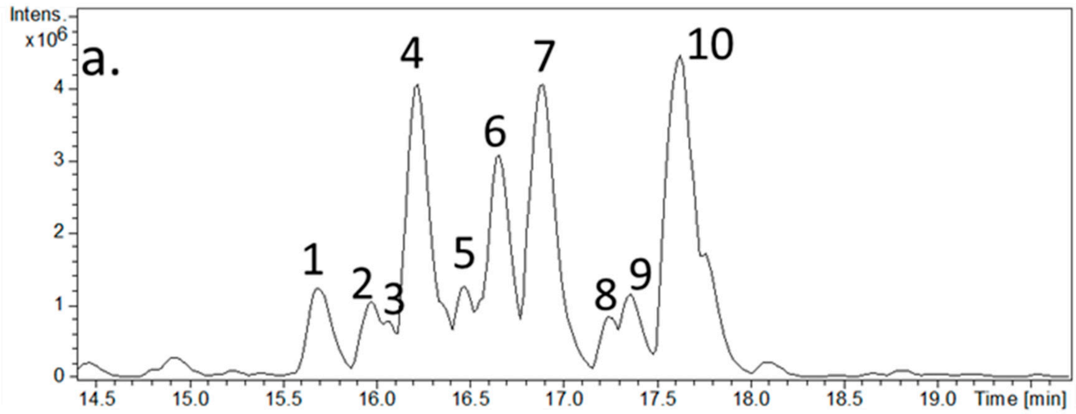

Supplemental Figure S3. Chromatogram of alkaloids present in fraction 2, which corresponds to R_t from 13.25 – 15.75 minutes of crude extract chromatogram.

The ten distinct peaks from fraction 2 have some overlap in retention times with fraction 1. Peak 1 has an m/z of 574.3425 and an elution time of 15.7 minutes, which corresponds to peak 2 of fraction 1 (**Figure S2**). Peak 2 has an m/z of 572.3264 and elution time of 16.0 minutes, which is the same as peak 3 from fraction 1 (**Figure S2**), which was predicted to be an isomer of veratrosine. Peak 3 from fraction 2 with an m/z of 576.3573, an elution time of 16.1 minutes, and a predicted molecular formula of $C_{33}H_{49}NO_7$ (identity unknown) does not have a peak corresponding to fraction 1. Neither does peak 4 with an m/z of 430.3073, elution time of 16.2 minutes, and predicted molecular formula of $C_{27}H_{43}NO_3$. This molecular formula is consistent with tetrahydrojervine.[4] Peak 5 with an m/z of 574.3424 and an elution time of 16.5 minutes matches that of peak 4 from fraction 1 (**Figure S2**). Thus, this peak is also predicted to be an isomer of cycloposine. Peak 6 had an m/z of 618.3671, an elution time of 16.6 minutes, and a predicted molecular formula of $C_{35}H_{55}NO_8$ (identity unknown). Peak 7 with an m/z of 578.3745, elution time of 16.9 minutes, and predicted molecular formula of $C_{33}H_{55}NO_7$ (identity unknown). Peak 8, which had an m/z of 472.3148, elution time of 17.2 minutes, and predicted molecular formula of $C_{29}H_{45}NO_4$ (identity unknown). Peak 9 had an m/z of 620.3871, an elution time of

17.3 minutes, and a predicted molecular formula of $C_{35}H_{57}NO_8$ (identity unknown). Finally, peak 10 had an m/z of 410.2828, an elution time of 17.6 minutes, and a predicted molecular formula of $C_{27}H_{39}NO_2$, which was confirmed to be veratramine.

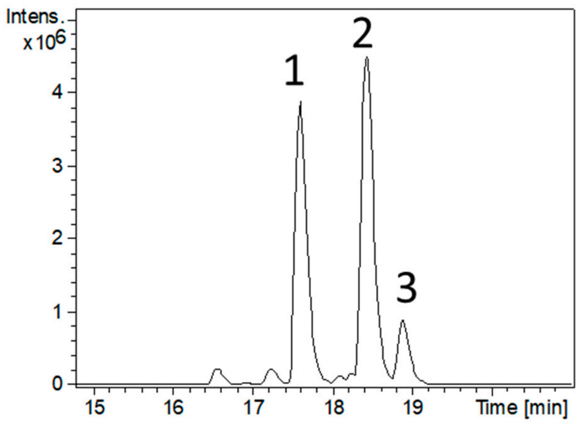

Supplemental Figure S4. Chromatogram of alkaloids present in fraction 3, which corresponds to Rt from 15.75 – 18.25 minutes of crude extract chromatogram.

Fraction 3 contains three alkaloids, of which only peak 10 from fraction 2 (veratramine) (**Figure S3)**, corresponds to peak 1 in fraction 3 with m/z ratios of 410.2828 and 410.3057 respectively. The elution time for this alkaloid is consistent between fractions at 17.6 minutes, lending further support for the identification. Peak 2 has an m/z of 412.3216, elution time of 18.4 minutes, and predicted molecular formula $C_{27}H_{41}NO_2$, which was confirmed to be cyclopamine. Peak 3 with an m/z of 412.3206, elution time of 18.9 minutes, and a predicted molecular formula of $C_{27}H_{41}NO_2$ (predicted to be cyclopamine's isomer[53]).

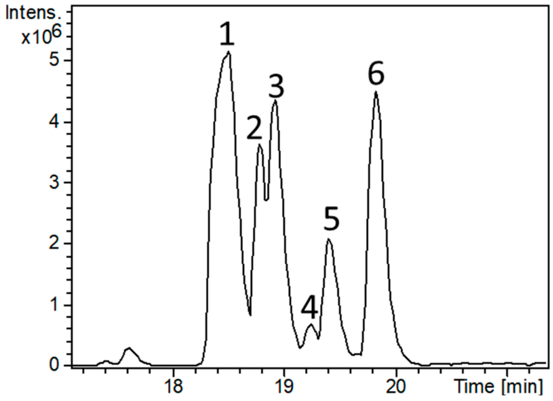

Supplemental Figure S5. Chromatogram of alkaloids present in fraction 4, which corresponds to R_t from 18.25 – 20.75 minutes of crude extract chromatogram.

Peak 1 is the first of the six alkaloids identified in fraction 4, and it has an m/z of 412.2996, elution time of 18.4 minutes, and predicted molecular formula $C_{27}H_{41}NO_2$, which is consistent with cyclopamine. Peak 2 has an m/z of 410.2815, elution time of 18.8 minutes, and predicted molecular formula of $C_{27}H_{39}NO_2$, which may be an isomer of veratramine. Peak 3 has an m/z of 412.2971, an elution time of 18.9 minutes, and a predicted molecular formula of $C_{27}H_{41}NO_2$, which is potentially an isomer of cyclopamine.[53] Peak 4 has an m/z of 474.3293, elution time of 19.2 minutes, and predicted molecular formula of $C_{29}H_{47}NO_4$ (identity unknown). Peak 5 has an m/z of 456.3208, an elution time of 19.4 min, and a predicted molecular formula of $C_{29}H_{45}NO_3$ (identity unknown). Lastly, peak 6 has an m/z of 458.3368, an elution time of 19.8 min, and a predicted molecular formula of $C_{29}H_{47}NO_3$, which is consistent with muldamine[4].

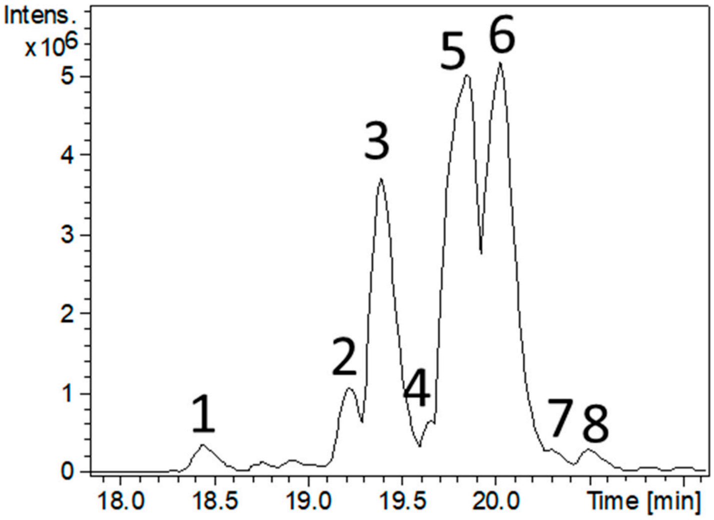

Supplemental Figure S6. Chromatogram of alkaloids present in fraction 5, which corresponds to R_t from 20.75 – 23.25 minutes of crude extract chromatogram.

Peak 1 from fraction 5 has an m/z of 412.2931, an elution time of 18.5 minutes, and a predicted molecular formula of $C_{27}H_{41}NO_2$, again corresponding to cyclopamine. Peak 2 has an m/z of 474.3302, an elution time of 19.2 minutes, and a predicted molecular formula of $C_{29}H_{47}NO_4$. This is consistent with peak 4 from fraction 4 (**Figure S5**), which has an unknown identity. Peak 3 has an m/z of 456.3214, an elution time of 19.4 minutes, and a predicted molecular formula of $C_{29}H_{45}NO_3$, which matches peak 5 from fraction 4 (**Figure S5**) but has an unknown identity. Peak 4 has an m/z of 416.3273, an elution time of 19.6 minutes, and a predicted molecular formula of $C_{27}H_{45}NO_2$, which has a suspected identity of 22-keto-26-aminocholesterol[4]. Peak 5 has an m/z of 458.3381, an elution time of 19.8 minutes, and a predicted molecular formula of $C_{29}H_{47}NO_3$. This peak matches peak 6 from fraction 4 (**Figure S5**), which was predicted to be muldamine. Peak 6 has an m/z of 416.3286, an elution time of 20.0 minutes, and a predicted molecular formula of $C_{27}H_{45}NO_2$. This predicted molecular formula is the same as that of peak 4 which is consistent with that of 22-keto-26-aminocholesterol[4]. Peak 7 has an m/z of 398.3171, an elution time of 20.3 minutes, and a

predicted molecular formula of $C_{27}H_{43}NO$, which is consistent with verazine[4]. The final peak, peak 8, has an m/z of 458.3354, an elution time of 20.5 minutes, and a predicted molecular formula of $C_{29}H_{47}NO_3$, which is representative of an isomer of muldamine.

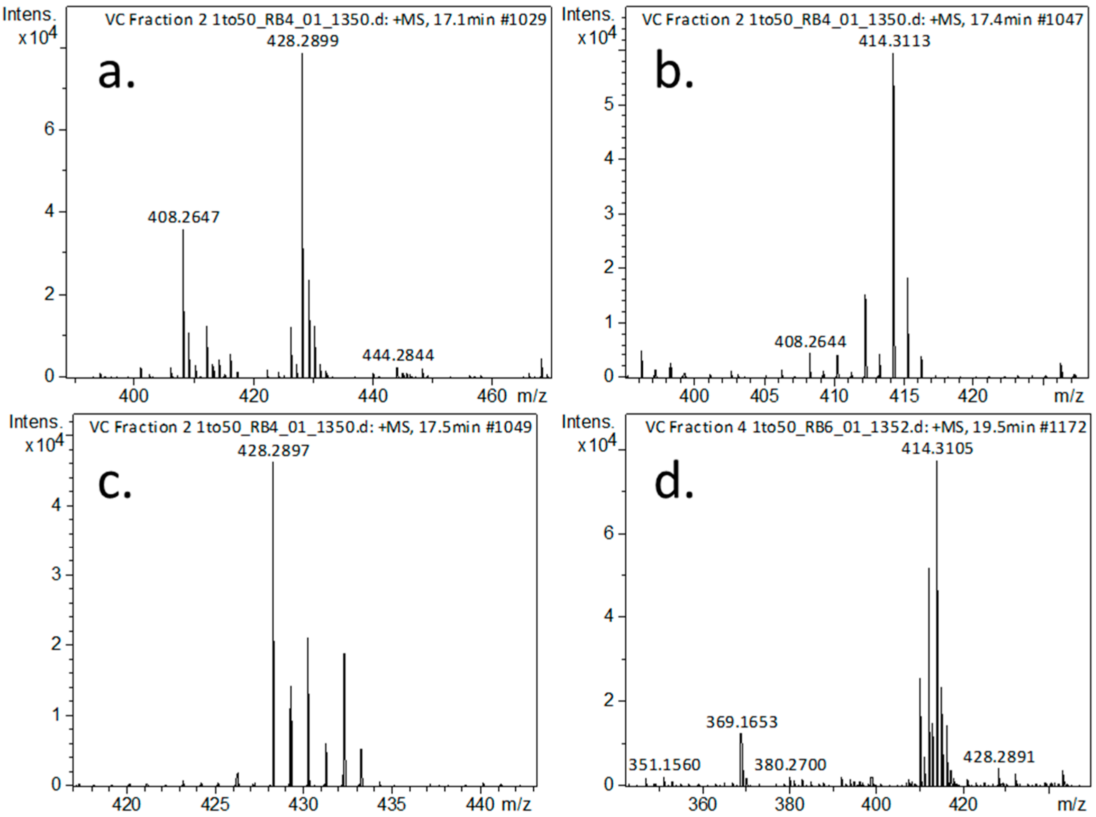

Supplemental Figure S7. Extracted ion chromatograms of previously identified alkaloids present in fractions 2 and 4 of crude alkaloid extract.

Figure S7 contains the alkaloids missing from the *V. californicum* extract as described in the literature.[4] The peak seen in **Figure S7 a.** came from fraction 2, has an m/z of 428.2899, an elution time of 17.1 minutes, and a predicted molecular formula of $C_{27}H_{41}NO_3$, representing dihydrojervine. **Figure S7 b.** shows a peak from fraction 2 with an m/z of 414.3113, an elution time of 17.4 minutes, and a predicted molecular formula of $C_{27}H_{43}NO_2$ corresponding to etioline. The peak seen in **Figure S7 c.** is also from fraction 2, has an m/z of 428.2897, an elution time of 17.5 minutes, and a predicted molecular formula of $C_{27}H_{41}NO_3$, which is consistent with dihydrojervine, and is thus assumed to be its isomer. The final peak in **Figure S7 d.** was from fraction 4, has an m/z of 414.3105, an elution time of 19.5 minutes, and a predicted molecular formula of $C_{27}H_{43}NO_2$, which is isorubijervine.

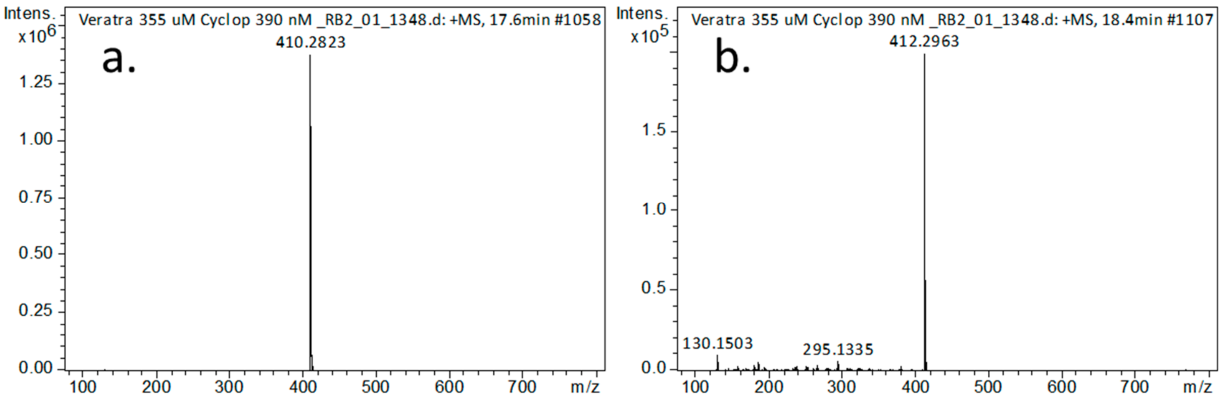

Supplemental Figure S8. Extracted ion chromatograms for veratramine and cyclopamine standards.

The peak in **Figure S8 a.** has an m/z of 410.2823, an elution time of 17.6 minutes, and a predicted molecular formula of $C_{27}H_{39}NO_2$, confirming its identity of veratramine. **Figure S8 b.** has an m/z of 412.2963, an elution time of 18.4 minutes, and a predicted molecular formula of $C_{27}H_{41}NO_2$, verifying the presence of cyclopamine.

The m/z ratios for commercial standards of veratramine and cyclopamine were 410.2823 and 412.2963 atomic mass units (amu), respectively (**Figure S8**), but the expected m/z ratios were about 55 amu lower than those values. This was likely due to the sample concentration exceeding the limit of detection, and thus all the alkaloid molecular formulas were generated with a margin of error within the range of 54.5-61.8 amu. Fraction 3 was an exception, as it was diluted by an additional order of magnitude, resulting in an error of less than one amu for veratramine and less than 2 amu for cyclopamine.
